# Metabolomics and gut microbiota analysis reveal the differential efficacy of areca nut and charred areca nut in treating constipation

**DOI:** 10.3389/fnut.2024.1455824

**Published:** 2024-09-13

**Authors:** Li-sha Wang, Jiao-xia Wu, Fang Zhang, Yan Huang, Yue-xia Jiang, Yong-hui Li

**Affiliations:** Key Laboratory of Tropical Translational Medicine of Ministry of Education, Hainan Provincial Key Laboratory of Research and Development on Tropical Herbs, Haikou Key Laboratory of Li Nationality Medicine, The Second Affiliated Hospital of Hainan Medical University, School of Pharmacy, Hainan Medical University, Haikou, China

**Keywords:** areca nut, charred areca nut, constipation, gut microbiota, metabolomics

## Abstract

**Background:**

Areca nut (AN) is a traditional Chinese herbal medicine used for centuries to treat gastrointestinal (GI) disorders. Charred AN (CAN) is a processed product of AN with similar therapeutic effects. This study aimed to investigate the therapeutic mechanisms of AN and CAN for constipation via metabolomics and gut microbiota analysis.

**Methods:**

In this study, the rats were randomly divided into 5 groups (*n* = 6): control, constipation model, positive drug, AN treatment, and CAN treatment groups. Constipation was induced by intragastric administration of loperamide hydrochloride, followed by 14-day treatment with mosapride, AN, or CAN. The efficacy difference between AN and CAN was assessed by evaluating the weight gain, fecal water content, GI transit rate, colonic histopathology, serum levels of GI hormones, gut microbiota, and fecal metabolites.

**Results:**

The results demonstrated that both AN and CAN could alleviate loperamide-induced constipation. Furthermore, they significantly elevated the serum levels of motilin, vasoactive intestinal peptide, substance P, and acetylcholine. 16S rRNA analysis revealed that AN regulated the relative abundance of *Bacillus*, *UCG-005*, *norank_f_Muribaculaceae*, *Candidatus_Saccharimonas*, and *Ruminococcus*, whereas CAN modulate the relative abundance of *Lactobacillus*, *Bacillus*, *norank_f_Muribaculaceae*, *Ruminococcus*, *unclassified_f_Oscillospiraceae*, and *unclassified_f_Prevotellaceae*. Moreover, the metabolic profile of AN- and CAN-treated rats was also different, where AN treatment involved pathways of citrate cycle (TCA) and tyrosine, alanine, aspartate, and glutamate metabolisms. Whereas CAN treatment involved pathways of steroid and primary bile acid biosynthesis, as well as pyrimidine and purine metabolisms. Spearman correlation analysis indicated a close relationship between gut microbiota and fecal metabolites.

**Conclusion:**

In summary, this study revealed that AN may protect GI mucosa, enhance GI motility, and alleviate constipation symptoms by regulating the relative abundance of specific gut microbiota (*Bacillus*, *UCG-005*, *norank_f_Muribaculaceae*, *Candidatus_Saccharimonas*, *Ruminococcus*) as well as citrate cycle or tyrosine, alanine, aspartate, and glutamate metabolic pathways. Furthermore, CAN was observed to promote gastric emptying and intestinal propulsion, thereby alleviating constipation, by modulating the relative abundance of specific gut microbiota (*Lactobacillus*, *Bacillus*, *norank_f_Muribaculaceae*, *Ruminococcus*, *unclassified_f_Oscillospiraceae*, *unclassified_f_Prevotellaceae*) as well as steroid and primary bile acid biosynthesis, as well as pyrimidine and purine metabolic pathways.

## Graphical abstract

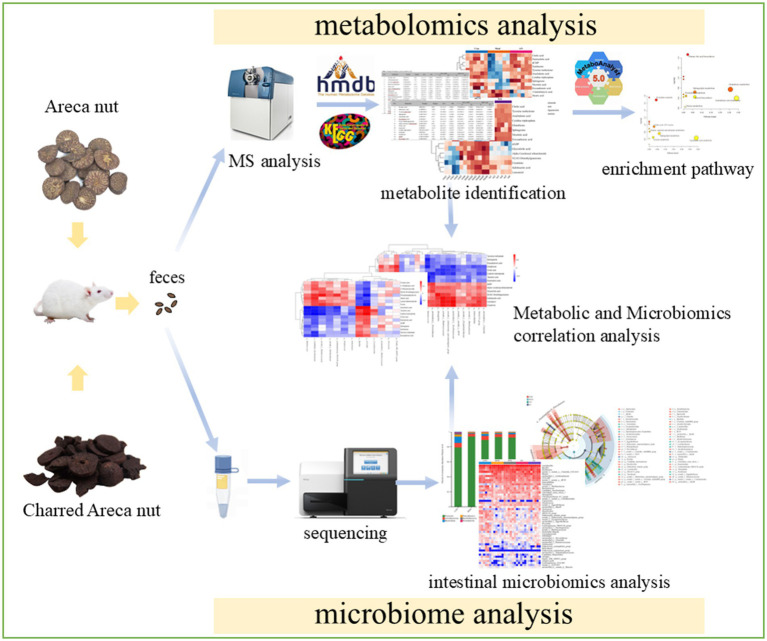

## 1 Introduction

Constipation is a common gastrointestinal (GI) disease with a global prevalence of about 15% in adults (1) and 15–50% in older individuals (2). It is characterized by reduced bowel frequency (<3 bowel movements/week), incomplete evacuation, dry stool, and anorectal obstruction (3). Prolonged constipation can cause abdominal spasms and worsen defecation difficulties, which can markedly impact a patient’s quality of life (4) and increase the risk of other GI diseases, such as intestinal inflammation, irritable bowel syndrome, and colorectal cancer (5, 6). Constipation is predominantly caused by colonic sensory motor disorders and pelvic floor dysfunction (3). The literature has indicated that loperamide can induce constipation, thereby substantially reducing intestinal transit rate and fecal water content (7). Therefore, because of its effects on GI motility, loperamide is the most commonly used drug in research studies for establishing a constipation model.

Areca nut (AN) is the dried seed of *Areca catechu* L., primarily cultivated in Hainan Province of China, and its production ranked 1st among China’s 4 famous southern medicines. It has been observed that AN can improve the GI function of functional dyspepsia rats and promote GI peristalsis by increasing GI smooth muscle contraction (8). Charred areca nut (CAN) is a processed product of AN, which is also utilized to increase digestive and absorptive functions, ameliorate gastric motility and emptying, and alleviate constipation (9). Furthermore, AN and CAN are the two widely used folk medicinal forms of betel nut and have been reported in the 2020 edition of the Chinese pharmacopoeia (10). Moreover, it has been identified that arecoline is the primary active compound in AN, and its effect is similar to that of muscarinic acetylcholine receptor agonists, which exerts cholinergic effects on the parasympathetic nervous system (11, 12). Arecoline acts on the M receptors to stimulate GI smooth muscle contraction, ameliorate GI motility, and enhance the GI tract’s digestive and absorptive activities (13). Therefore, arecoline in AN and CAN is considered a bioactive component that can be employed to improve GI functions (14, 15). However, it has been observed that the content of arecoline is significantly reduced in processed CAN (16), whereas GI motility increased after CAN intervention. This suggests that arecoline may not be the predominant component related to the different efficacy of AN and CAN in the treatment of constipation. Therefore, the mechanisms underlying the efficacious effects of CAN and AN on constipation warrant further research.

Clinical research studies have indicated that alterations in the gut microbiota are associated with constipation. The gut microbiota participates in various physiological functions in the host, such as modulating intestinal motility and barrier function (17). Several studies have indicated that the gut microbiota’s community structure between healthy and constipated individuals is significantly different (18–21). Furthermore, for normal gut functioning, the host’s gut microbiota should be at homeostasis (22). Disturbing the dynamic balance of intestinal flora can disrupt human metabolism, causing various pathophysiological changes and aggravating constipation symptoms (23).

Gut microbiota metabolites are identified by abundant chemosensors in the intestine and influence intestinal motility and secretion, contributing to intestinal physiological function and environmental stability (24). Therefore, intestinal metabolite alterations are also associated with constipation. It has been reported that the microbiome and metabolome could interact with each other; that is, the gut microbiota can affect metabolite composition, and the metabolites can shape the structure of the microbial community (25). Studies have shown that fecal metabolomics can be used to assess the effects of gut microbes of a constipation patient on the gut metabolic environment and can also reflect the state of constipation (26). Therefore, omics studies of intestinal metabolites are important for discovering the internal mechanism of intestinal dysfunction.

This study analyzed the differences in the efficacy of AN and CAN for the treatment of constipation via gut microbiota and fecal metabolomics. This research aims to highlight the potential mechanisms of AN and CAN in treating constipation and provide a better understanding of efficacy differences between AN and CAN.

## 2 Materials and methods

### 2.1 Reagent

The loperamide hydrochloride was purchased from Xi’an Yangsen Pharmaceutical Co., Ltd. (China). Mosapride was acquired from Yabao Pharmaceutical Group Co., Ltd. (China). Acetonitrile, methanol, and formic acid (chromatography pure) were procured from Merck (Germany). AN and CAN were obtained from Hainan Linshi Shengtai Pharmaceutical Co., Ltd. (Haikou, China).

### 2.2 Water extracts of AN and CAN

AN and CAN (100 g, respectively) were crushed, soaked in water (1/10, w/v) for 1 h, and then subjected to heat reflux extraction for 1 h, respectively. Then, the extracted CAN and AN liquids were concentrated to 100 mL under reduced pressure to acquire their extracts.

### 2.3 Animal experimental design

Male SD rats (weight = 180–220 g, *n* = 30) were purchased from Changsha Tianqin Biotechnology Co., Ltd. (Certificate No.: CSXK Xiang 2022-0011) and provided *ad libitum* access to standard feed and water. All the animal analyses were authorized by the Ethics Committee of Hainan Medical University (Approval No.: HYLL-2021-308) and followed the guidelines for the care and use of laboratory animals.

All the rats were randomly divided into 5 groups (*n* = 6/group), including control (CON), constipation model (MOD), positive drug (POS), AN treatment (AN), and CAN treatment (SA) groups. The constipation model was established by intragastric administration of loperamide (8 mg/kg). The CON group was given physiological saline. The POS group was administered loperamide to induce constipation and then intragastrically treated with mosapride (2 mg/kg). The AN and SA group rats were administered loperamide and then intragastrically treated with AN (3 mL/kg) and CAN (3 mL/kg) extract, respectively. The treatment lasted for 14 days, and the weight gain of each group’s rats was assessed daily. On the last day of treatment, fecal pellets of all the rats were collected within 24 h and weighed. Dry feces were acquired by collecting and drying fresh feces in an oven at 60°C for 24 h. The fecal water content of rats was calculated using the following formula:


$$\begin{array}{l} \mathrm{Fecal}\;\mathrm{water}\;\mathrm{content}\;\left(\%\right)\\ =\left[\begin{array}{l} \left(\mathrm{fresh}\;\mathrm{fecal}\;\mathrm{weight}-\mathrm{dry}\;\mathrm{fecal}\;\mathrm{weight}\right)\\ /\mathrm{fresh}\;\mathrm{fecal}\;\mathrm{weight} \end{array}\right]\times 100\% \end{array}$$

To measure their GI transit rate, all the rats were fasted for 12 h after the last treatment and then received a 10% carbon ink via gavage. The GI transit rate of the carbon ink was calculated using the following equation:


$$\begin{array}{l} GI\;\mathrm{transit}\;\mathrm{rate}\;\left(\%\right)\\ =\left(\begin{array}{l} \mathrm{distance}\;\mathrm{of}\;\mathrm{carbon}\;\mathrm{powder}\;\mathrm{movement}/\\ \mathrm{total}\;\mathrm{length}\;\mathrm{of}\;\mathrm{small}\;\mathrm{intestine} \end{array}\right)\times 100\%\text{.} \end{array}$$

Rat serum was collected by centrifuging their blood at 3,000 rpm for 10 min at 4°C, which was then stored at −80°C for further analysis. Colon tissue and fecal were also collected for further analyses.

### 2.4 Histological evaluation

Briefly, the rat’s colon tissues were fixed with 4% polyformaldehyde, dehydrated, paraffin-embedded, sectioned (4 μm thick), stained with hematoxylin and eosin (HE), and imaged under a light microscope.

### 2.5 Detection of motilin, vasoactive intestinal peptide, substance P, and acetylcholine

The rat serum levels of motilin (MTL), vasoactive intestinal peptide (VIP), substance P (SP), and acetylcholine (ACh) were detected using an ELISA kit from Nanjing Jiancheng Bioengineering Institute (Nanjing, China), per the kit’s instructions.

### 2.6 Intestinal microbiota 16S rRNA gene sequencing

The rat’s cecum contents were collected for gut microbiota analysis. Total microbial genomic DNAs were extracted using the E.Z.N.A.^®^ Stool DNA Kit (Omega Bio-tek, Norcross, GA, United States) according to the manufacturer’s instructions. Then, the DNA’s quality and concentration were assessed via 1.0% agarose gel electrophoresis and a NanoDrop2000 spectrophotometer (Thermo Scientific, United States), respectively. The bacterial 16S rRNA gene’s hypervariable region V3–V4 was amplified with primer pairs 338 F (5′-ACTCCTACGGGAGGCAGCAG-3′) and 806 R (5′-GGACTAChVGGGTWTCTAAT-3′) (27) using the T100 Thermal Cycler PCR (BIO-RAD, United States). Subsequently, PCR products were recovered using 2% agarose gel, purified with PCR Clean-Up Kit (YuHua, Shanghai, China), and quantified via Qubit 4.0 (Thermo Fisher Scientific, United States). The purified amplicons were pooled at equimolar amounts on the Illumina PE300 platform (Illumina, San Diego, United States) for paired-end sequencing, which was performed by following the standard protocol from Majorbio Bio-Pharm Technology Co.

### 2.7 Analysis of intestinal flora data

Bioinformatic analysis of the gut microbiota was carried out using the Majorbio Cloud platform.^1^ The gut microbiota analysis was performed using Qiime2 (version 2020.2) and the SILVA 16S rRNA database (v138). Based on different similarity levels, all sequences were classified into ASVs, which were then subjected to bioinformatics analysis with a 97% similarity level. Mothur software (version 1.30) was employed for alpha diversity analysis based on the ASV sequences. The inter-group differences in alpha diversity data were tested via one-way ANOVA. For beta diversity analysis, non-metric multidimensional scaling analysis (NMDS) was performed. Based on the results of taxonomic analysis, R language (version 3.3.1) was used to analyze the community structure composition of different groups at the phylum level. The similarities and differences of community composition at the genus level were analyzed by heatmap, drawn using the R language (version 3.3.1) pheatmap (1.0.8) package. The non-parametric Kruskal–Wallis (KW) rank-sum test was carried out for Linear discriminant analysis Effect Size (LEfSe) analysis to detect differences in species abundance between different taxa and obtain significantly different species. Moreover, the Wilcoxon rank-sum test was performed to evaluate consistency between different species in different subgroups of the previous different taxa. In addition, linear discriminant analysis (LDA) was conducted to estimate the effect of these species on the differences between groups.

### 2.8 Metabolite analysis via UPLC-Q-TOF/MS

The fecal samples at −80°C were removed from the refrigerator and slowly thawed at 4°C. Then, 200 mg of feces was transferred in a 2 mL centrifuge tube containing 800 μL methanol, vortexed for 2 min, sonicated in an ice bath for 20 min, and centrifuged (13,000 rpm) at 4°C for 15 min to collect the supernatant, which was then filtered through a 0.22 μm microporous membrane before test.

Rat fecal metabolomics analysis was performed using a high-resolution mass spectrometer (AB Sciex Triple TOF 4600) coupled with a UHPLC system (Agilent 1290, United States) in positive and negative ion modes. The chromatographic column was Waters Acquity UPLC HSS T3 (1.8 m, 2.1 × 100 mm). The chromatographic mobile phase was acetonitrile (A) and 0.1% formic acid (B). The gradient elution conditions were: 0–1 min of 5% A, 1–15 min of 5–95% A, 15–19 min of 95% A, 19–20 min of 95–5% A, and 20–22 min of 5% A. The column temperature was 35°C, the flow rate was set to 0.3 mL/min, and the injection volume was 3 μL. The mass spectrometry conditions were: positive or negative ion modes were used for ESI ion source detection, mass detection range = *m*/*z* 100–1,000 Da, source temperature = 550°C, ion spray voltage = +5,500/−4,500 V, declustering potential (DP) = ±80 V, collision energy = ±35 eV, curtain gas = 30 psi, and ion source gas 1 and 2 = 50 psi.

### 2.9 Data processing and analysis

The peak identification, extraction, and area normalization of raw mass spectrometry data was performed on Markview 1.3.1 (AB Sciex) software (28). For multivariate statistical analysis, SIMCA 14.1 (Umetrics, Umea, Sweden) was employed. The model’s veracity and predictive capabilities were assessed using R2 Y and Q2 parameters. Different metabolites were screened based on the following criteria: VIP >1, *p* < 0.05, FC >1.2, or FC <0.8 (29). Peakview 1.2.1 software was used to screen potential metabolites by secondary mass spectrometry, then identified by HMDB^2^ and KEGG.^3^ Moreover, the identified metabolite’s metabolic pathway enrichment analysis was performed using MetaboAnalyst 5.0^4^ (30). Spearman correlation analysis was performed to assess the relationship between metabolites and gut microbiota.

### 2.10 Statistical analyses

GraphPad Prism 5.01 software was employed for all statistical assessments, and the data are expressed as mean ± SD. Student’s *t*-tests were performed to assess the statistical differences, and *p* < 0.05 was termed considered statistically significant.

## 3 Results

### 3.1 Body weight gain, fecal water content, and GI transit rate of rats

**Figure 1:**
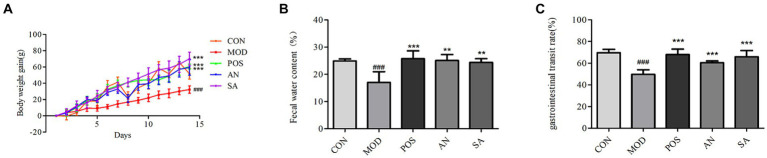
Effects of AN and CAN on loperamide-induced constipation in rats. **(A)** Daily body weight of rats. **(B)** Fecal water content. **(C)** Gastrointestinal transit rate. Data are displayed as mean ± SD. ^#^*p <* 0.05, ^##^*p <* 0.01, and ^###^*p <* 0.001 vs. CON group. ^**^p < 0.01 and ^***^p < 0.001 vs. MOD group.

To assess the effects of AN and CAN on weight gain in constipated rats, their weight was recorded daily (Figure 1A), which gradually increased in all the groups during the experimental period. However, the weight gain of constipated rats was significantly lower than that of normal rats (*p* < 0.001). Furthermore, the MOD rat’s weight was significantly lower than that of the POS (*p* < 0.001), AN group (*p* < 0.001) and SA (*p* < 0.001) rats.

Dry stool is common in constipation, and the water content of feces can be used to determine the successful establishment of the constipation model and the severity of constipation. Increased fecal water content indicates the increase of intestinal peristalsis, which could relieve symptoms of constipation. The fecal water content of MOD rats was decreased (17.01 ± 3.99) on the 14th day of constipation induction, which was significantly lower than that in the CON rats (24.88 ± 0.68, *p* < 0.05) (Figure 1B). However, after treatment, the feces moisture content of rats in the POS (25.75 ± 2.89), AN (25.09 ± 2.19), and SA (24.39 ± 1.35) groups was significantly increased compared to the MOD group (*p* < 0.05).

GI transit rate directly reflects GI motility. The intestinal transit rates for the CON, MOD, POS, AN, and SA groups were 69.74 ± 3.09, 49.75 ± 4.12, 67.98 ± 5.06, 60.41 ± 1.61, and 65.85 ± 5.87, respectively (Figure 1C). After AN and CAN interventions, the GI transit rate increased significantly compared with the MOD group (*p* < 0.05). Moreover, no significant differences were observed between GI motility in AN, SA, and POS groups, indicating that AN and CAN could promote GI motility.

### 3.2 Histopathological evaluation of rat colon tissue

**Figure 2:**
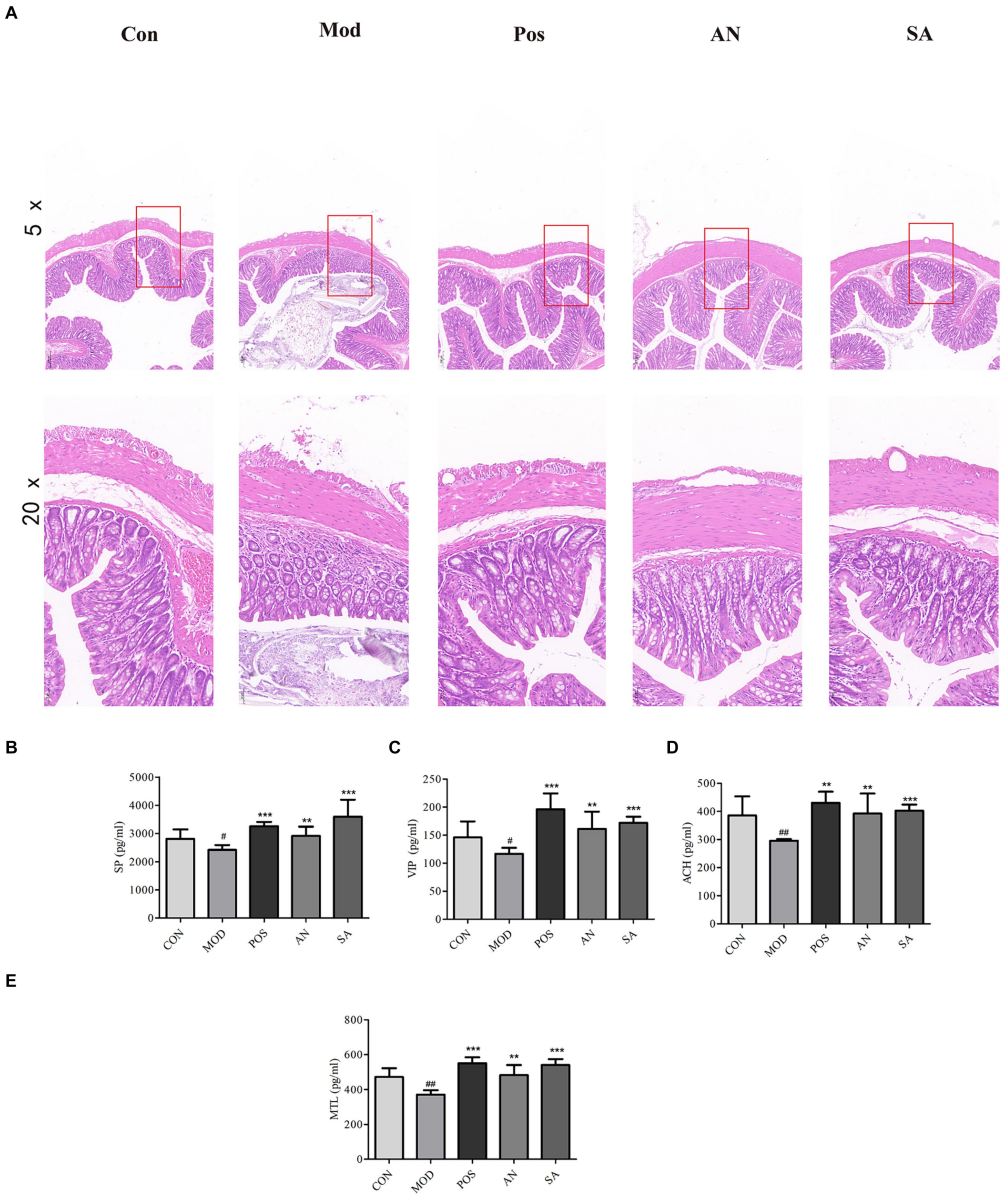
H&E staining of rat’s colon tissues (magnification = 5× and 20×). **(A)**. Levels of substance P (SP) **(B)**, vasoactive intestinal peptide (VIP) **(C)**, acetylcholine (ACh) **(D)**, and motilin (MTL) **(E)** in rat serum. Data are displayed as mean ± SD. ^#^*p <* 0.05 and ^##^*p <* 0.01 vs. CON group. ^*^p < 0.05, ^**^p < 0.01, and ^***^p < 0.001 vs. MOD group.

The colon structure of CON rats was intact, with regularly arranged mucosal glands (Figure 2A). However, in the MOD group, the colon muscle layer had edema, crypts were atrophied, mucosal goblet cells were reduced, and there was abnormal inflammatory cell infiltration. After treatment with mosapride, AN, and CAN, the cryptic structure of the rat colon was restored, mucosal goblet cells increased, and muscle edema and abnormal inflammatory infiltrates were reduced. Overall, histological analysis indicated that AN and CAN effectively ameliorated intestinal barrier damage in constipated rats.

### 3.3 The effects of AN and CAN on serum GI hormone levels in rats

The serum levels of MTL, SP, VIP, and ACh in constipated rats were significantly reduced (Figures 2B–E); however, mosapride, AN, and CAN increased these levels.

### 3.4 The effects of AN and CAN on intestinal microbiota diversity in rats

To study the effects of AN and CAN on constipated rat’s gut microbiota as well as compare the changes in intestinal microbiota between the five groups, 16S rRNA gene sequencing analysis was performed. With an increase in sequencing depth, the rarefaction curves of the Shannon index gradually approached stability, with coverage exceeding 0.9937 (Supplementary Figures S1A,B), indicating sufficient sequencing data warranting further investigation. The total number of ASVs and unique ASVs in each group were visualized via Venn diagrams to assess ASV abundance across samples. It was revealed that each group had 117 ASVs, with 6 and 10 unique ASVs in the AN and SA groups, respectively, distinct from the CON, MOD, and POS groups (Supplementary Figure S1C).

Alpha diversity analysis was conducted to determine the richness and diversity of intestinal microbiota species (31). ACE and Chao indices indicated the species richness of each sample community. ACE was employed to assess a community’s exponential number of ASVs, while the Chao value increased with the number of ASVs in the sample. Furthermore, the Simpson index was utilized to assess the diversity of microorganisms in the sample, with higher index values indicating lower community diversity. Sobs represented the actual number of observed ASVs. The MOD group’s ACE and Chao indices were significantly lower than those of the CON group (*p* < 0.05) (Supplementary Figures S1D–G). These indices were substantially higher in the POS and SA groups than in the MOD group, indicating that CAN could alleviate the decrease in species richness caused by loperamide-induced constipation. Moreover, the Simpson index of loperamide-induced rats was significantly higher than the normal rats (*p* < 0.05). However, after CAN treatment, the Simpson index in the SA rats indicated a significant reverse trend, suggesting that CAN increased community diversity. Compared to the CON group, the Sobs index of the MOD group was significantly decreased (*p* < 0.05), while relative to the MOD group, the Sobs index of the POS and SA groups was significantly increased. In the Beta diversity analysis, the differences between groups were obtained by NMDS analysis and reflected as points in the multidimensional space. The data showed a significant spatial distance between the MOD and CON groups, indicating that loperamide markedly altered the rat’s intestinal microbial community (Supplementary Figure S1H). In addition, the mosapride, AN, and CAN-treated rats were spatially altered from constipated rats, indicating that these treatments altered the constipated rat’s bacterial community structure.

### 3.5 The impact of AN and CAN on the relative abundance of intestinal microbiota in rats

The community structure of gut microbiota was investigated at the phylum level. The data revealed that the intestinal microbiota of rats primarily comprised Firmicutes, Actinobacteria, Bacteroidetes, Patescibacteria, Desulfobacteria, and Proteobacteria (Supplementary Figure S2A). Among these, Firmicutes and Bacteroidetes were predominant phyla in healthy rats, and their ratio in CON rats was 5.29, which significantly increased to 315.46 after loperamide induction (Supplementary Figures S2B–D). However, the Firmicutes to Bacteroidota (F/B) ratio in the POS (F/B = 36.35), AN (F/B = 45.21), and SA (F/B = 44.74) groups was significantly decreased after the treatment (*p* < 0.05). Loperamide-induced constipation decreased the relative abundance of Bacteroidetes, which was significantly reversed in POS, AN, and SA groups. Compared to the CON group, loperamide increased the relative abundance of Proteobacteria and significantly decreased the relative abundance of Proteobacteria in the POS, AN, and SA groups.

**Figure 3:**
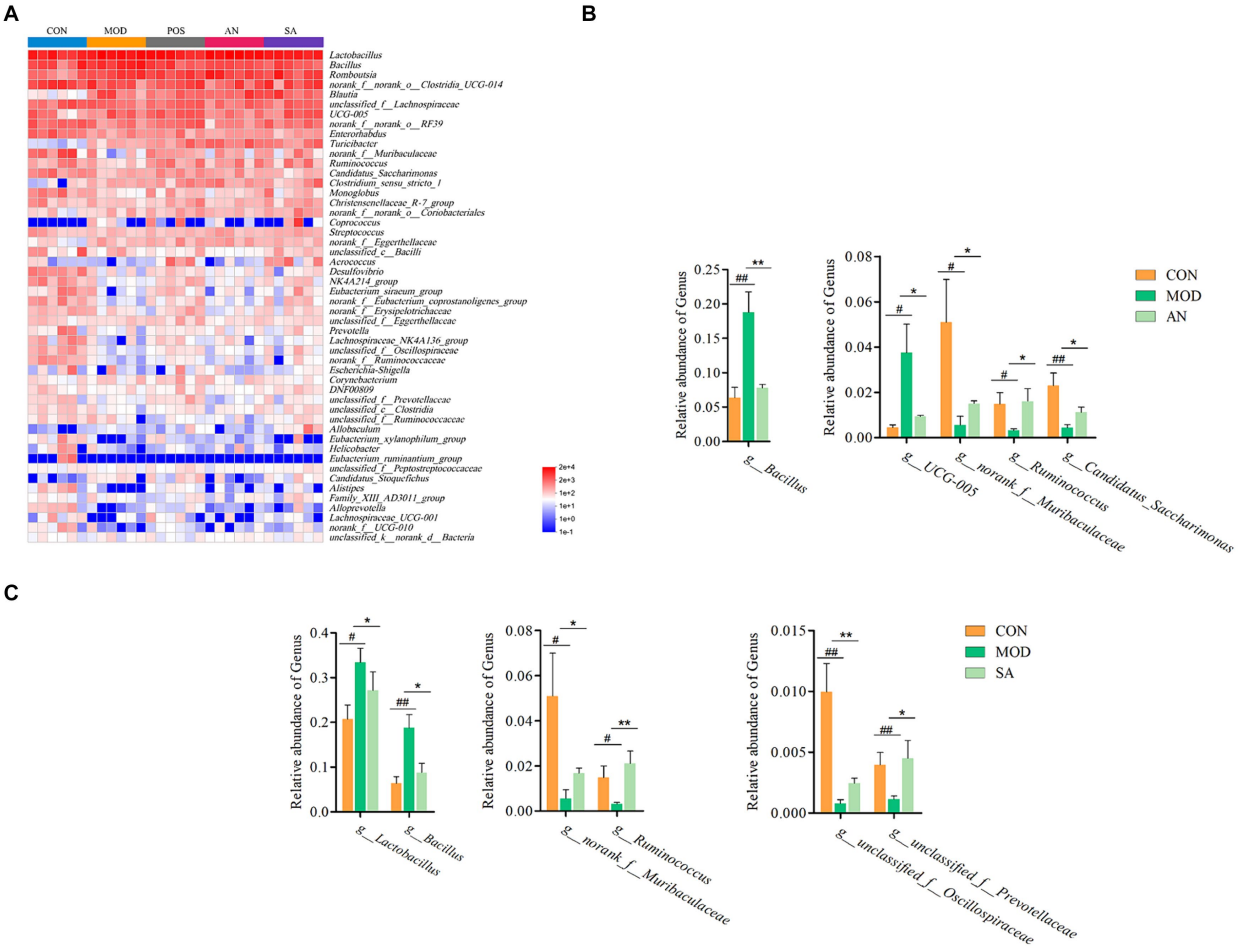
AN and CAN alter microbial composition at the genus levels. **(A)** Community heatmap at the genus level. **(B)** Relative abundance of *Bacillus*, *UCG-005*, *norank_f_Muribaculaceae*, *Ruminococcus*, and *Candidatus_Saccharimonas* before and after AN treatment. **(C)** Relative abundance of *Lactobacillus*, *Bacillus*, *norank_f_Muribaculaceae*, *Ruminococcus*, *unclassified_f_Oscillospiraceae*, and *unclassified_f_Prevotellaceae* before and after CAN treatment. Data are displayed as mean ± SD. ^#^*p <* 0.05, ^##^*p <* 0.01, and ^###^*p <* 0.001 vs. CON group. ^*^p < 0.05, ^**^p < 0.01, and ^***^p < 0.001 vs. MOD group.

Heatmap analysis was conducted on the intestinal microbiota of five groups of rats at the genus level (Figure 3A). It was observed that loperamide markedly altered the relative abundance of intestinal microbiota, which was reversed after AN and CAN treatments. AN treatment reversed the constipation-induced imbalance of relative abundance of *g_norank_f_Muribaculaceae*, *g_Candidatus_Saccharimonas*, *g_Bacillus*, *g_UCG-005*, and *g_Ruminococcus* (Figure 3B). Whereas CAN regulates the relative abundance changes of *g_Lactobacillus*, *g_Bacillus*, *g_norank_f_Muribaculaceae*, *g_Ruminococcus*, *g_unclassified_f_Oscillospiraceae*, and *g_unclassified_f_Prevotellaceae* (Figure 3C). These data revealed that loperamide-induced constipation disrupts the intestinal microbiota abundance at the genus level, which can be effectively improved by AN and CAN treatments, although these interventions modulate different microbiota.

**Figure 4:**
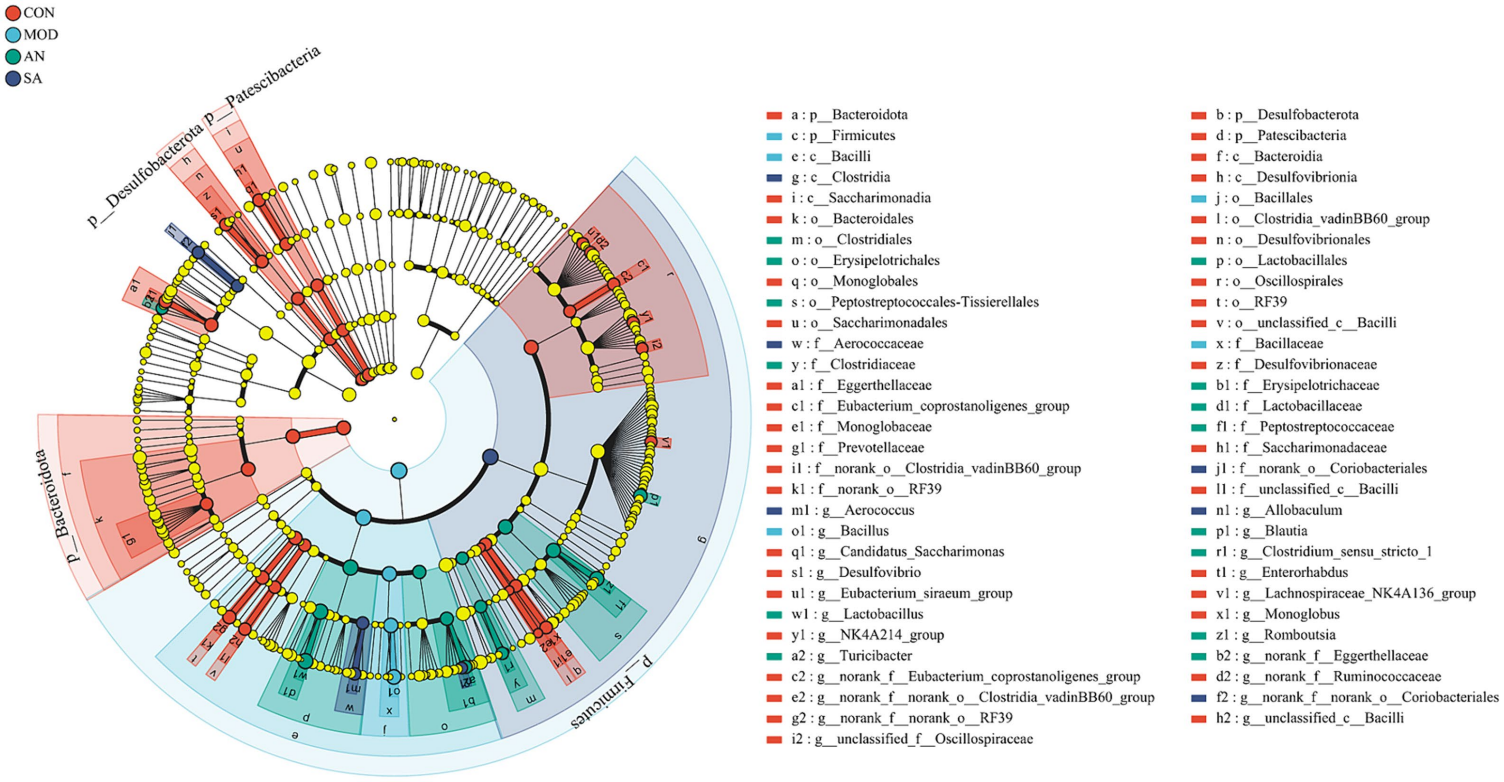
LEfSe analysis. LEfSe histograms at the genus, family, order, class, and phylum levels (LDA score >3.5).

The characteristic bacteria and biomarkers in each group were further identified by LEfSe analysis, which revealed significant differences in intestinal microbiota at various levels in different groups (Supplementary Figure S3, LDA score >3.5). At the phylum level (Figure 4), the predominant bacteria in the CON group were Bacteroidetes, Desulfobacteria, and Patescibacteria, while in the MOD group was Firmicutes. At the class level, the CON group biomarkers included Eggerthellaceae, Desulfovibrionaceae, Eubacterium_coprostanoligenes_group, Monoglobaceae, Provotellaceae, norank_o_Clostridia_vadinBB60_group, Saccharimonadaceae, norank_o_RF39, and unclassified_c_Bacilli. Whereas that of the MOD group was Bacillaceae. In addition, *g_norank_f_Eggerthellaceae*, *g_Lactobacillus*, *g_Turicibacter*, *g_Clostridium_sensu_stricto_1*, *g_Romboutsia*, and *g_Blautia* were identified as the main biomarkers for the AN group, potentially serving as specific targets modified by AN on the intestinal microbiota. For the SA group, *g_norank_f_norank_o_Coriobacteriales*, *g_Allobaculum*, *g_Aerococcus*, and c_Clostridia were identified as specific targets.

### 3.6 The influence of AN and CAN on the fecal metabolites of rats

To investigate the impact of AN and CAN on the intestinal metabolites of constipated rats, CON, MOD, AN, and SA rat’s fecal samples were subjected to ultra-high-performance liquid chromatography-quadrupole time-of-flight mass spectrometry (UHPLC-QTOF-MS) analysis in positive and negative ion modes. For multivariate statistical analysis of the fecal metabolite data, Principal component analysis (PCA) was performed to explore the differences between the groups. The PCA score plots (Supplementary Figures S4A,B) revealed that fecal samples from different groups of rats were all variable. Orthogonal partial least squares discriminant analysis (OPLS-DA) was performed on the CON and MOD rat’s fecal samples to further investigate the metabolic differences in constipated rats. Significant variability was observed between the MOD group and the CON, AN, and SA groups (Supplementary Figures S4C–H) in both positive and negative ion modes, with R2Y values of 1 and Q2 values >0.5. These results suggested the presence of metabolic abnormalities in the feces of constipated rats. The models showed no overfitting after 200 permutation tests, validating the reliability of the predictive models (Supplementary Figure S5).

Volcano plots were utilized to visualize differential metabolites between the groups in positive and negative ion modes. Compared with the CON rat, the MOD rat’s fecal samples indicated 407 upregulated and 548 downregulated metabolites in positive ion mode and 347 upregulated and 1994 downregulated metabolites in negative ion mode (Supplementary Figures S6A-F). Furthermore, compared to the MOD group, the AN group had 395 upregulated and 445 downregulated metabolites in positive ion mode and 88 upregulated and 3,060 downregulated metabolites in negative ion mode. In addition, the SA group exhibited 338 upregulated and 642 downregulated metabolites in positive ion mode and 188 upregulated and 3,218 downregulated metabolites in negative ion mode.

**Figure 5:**
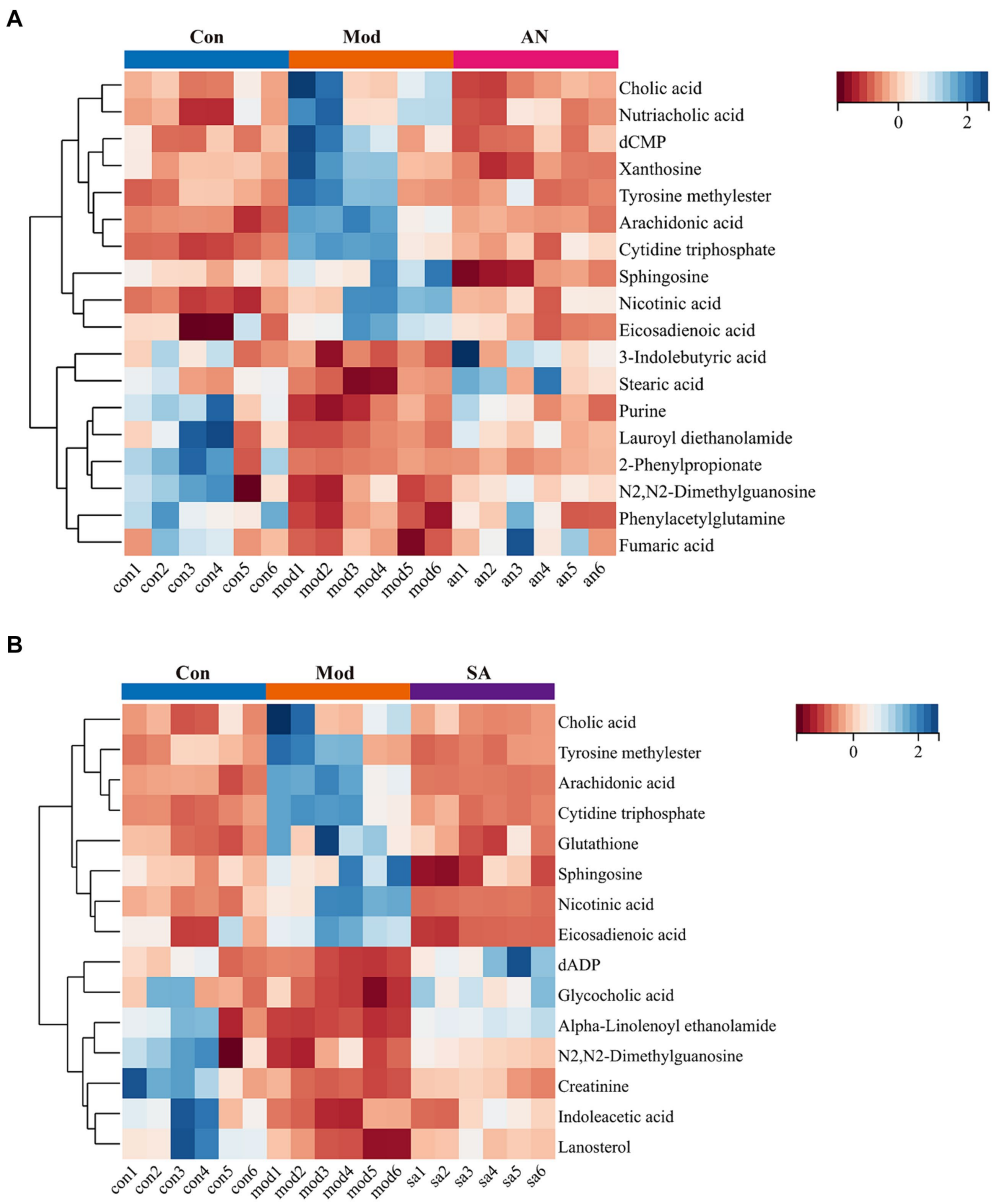
The expression of differential metabolites in **(A)** AN and **(B)** CAN rats.

**Figure 6:**
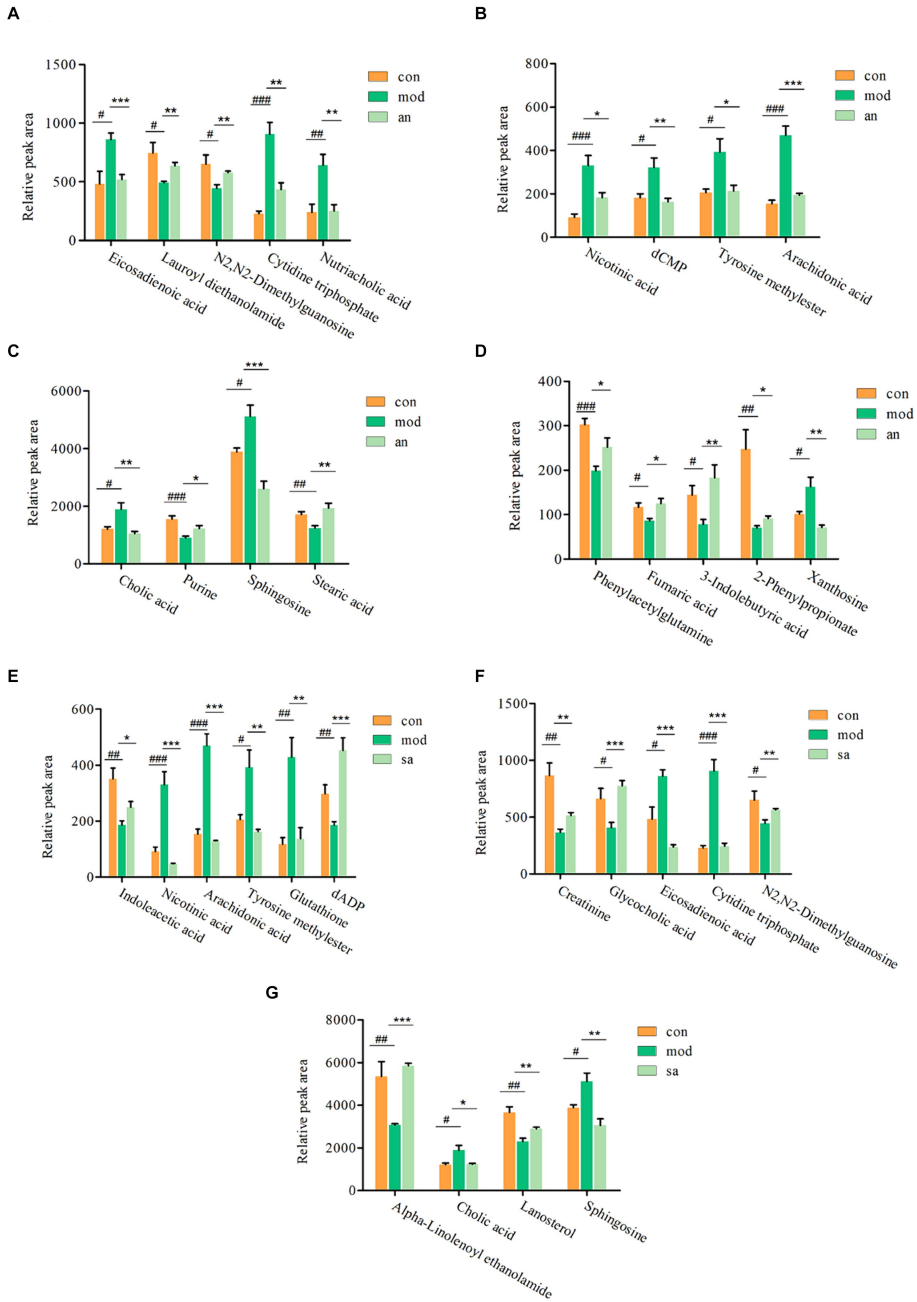
The ionic strength of potential metabolites in rat feces **(A–G)**. Data are displayed as mean ± SD. ^#^*p <* 0.05, ^##^*p <* 0.01, and ^###^*p <* 0.001 vs. CON group. ^*^p < 0.05, ^**^p < 0.01, and ^***^p < 0.001 vs. MOD group.

Based on VIP >1, *p* < 0.05, FC >1.2, or FC <0.8, common differential metabolites between the AN group and the CON or MOD groups, as well as between the SA group and the CON or MOD groups, were screened. The HMDB screening identified 18 differential metabolites between the AN and CON or MOD groups (Table 1), whereas 15 differential metabolites between the SA and CON or MOD groups (Table 2). Heatmap analysis was conducted to illustrate the expression of differential metabolites among the groups (Figures 5A,B), which revealed significant changes in metabolites between the CON and MOD groups, reversed by the AN and SA groups. In the CON, MOD, and AN groups, 18 metabolites were identified (Figures 6A–D). Compared to the CON group, nicotinic acid, tyrosine methylester, sphingosine, arachidonic acid, eicosadienoic acid, nutriacholic acid, cholic acid, cytidine triphosphate, xanthosine, and dCMP were significantly upregulated in the MOD group, while 2-phenylpropionate, phenylacetylglutamine, stearic acid, lauroyl diethanolamide, N2,N2-dimethylguanosine, fumaric acid, purine, and 3-indolebutyric acid were downregulated.

**Table 1:** Potential biomarkers in feces treated by AN.

No.	Metabolites	Formula	Rt (min)	*m*/*z*	VIP	CON vs. MOD		MOD vs. AN	
						*p*	FC	*p*	FC
1	Nicotinic acid	C_6_H_5_NO_2_	9.89	123.1175	1.67863	0.000608505	3.59901	0.0165418	0.552462196
2	2-Phenylpropionate	C_9_H_10_O_2_	7.85	150.0906	1.55819	0.00255877	0.284573	0.0174449	1.299396472
3	Tyrosine methylester	C_10_H_13_NO_3_	7.17	195.0636	1.33353	0.0159653	1.90626	0.0249652	0.541465468
4	Phenylacetylglutamine	C_13_H_16_N_2_O_4_	9.96	264.1111	1.76341	0.000132314	0.657634	0.0431869	1.266473959
5	Stearic acid	C_18_H_36_O_2_	12.64	284.2946	1.52157	0.00385537	0.724186	0.00354181	1.559251693
6	Lauroyl diethanolamide	C_16_H_33_NO_3_	13.20	287.3148	1.30722	0.019571	0.660325	0.0011393	1.292909334
7	Sphingosine	C_18_H_37_NO_2_	16.23	299.2932	1.37008	0.0143366	1.31612	0.000348656	0.509491134
8	Arachidonic acid	C_20_H_32_O_2_	6.79	304.1652	1.80389	4.04 × 10^−5^	3.04138	8.04506 × 10^−5^	0.412548322
9	Eicosadienoic acid	C_20_H_36_O_2_	0.73	308.2173	1.3962	0.0105847	1.78669	0.00067017	0.60325813
10	N2,N2-Dimethylguanosine	C_12_H_17_N_5_O_5_	5.62	311.1033	1.21087	0.0351331	0.681728	0.00409974	1.302499651
11	Nutriacholic acid	C_24_H_38_O_4_	10.74	390.0471	1.47313	0.00560662	2.67493	0.00444872	0.389909899
12	Cholic acid	C_24_H_40_O_5_	12.99	408.3122	1.32594	0.0170507	1.56328	0.00518554	0.554832929
13	Cytidine triphosphate	C_9_H_16_N_3_O_14_P_3_	0.90	483.1095	1.78379	6.73 × 10^−5^	3.97233	0.0023064	0.480240401
14	Fumaric acid	C_4_H_4_O_4_	16.54	115.9172	1.42007	0.016419	0.737177	0.0141164	1.447992691
15	Purine	C_5_H_4_N_4_	7.65	119.9156	1.79918	0.000415684	0.5827	0.02246	1.354086744
16	3-Indolebutyric acid	C_12_H_13_NO_2_	1.14	203.0214	1.38739	0.0200071	0.545382	0.00716489	2.326669762
17	Xanthosine	C_10_H_12_N_4_O_6_	7.91	284.1191	1.38386	0.0205031	1.60662	0.00220092	0.4375327
18	dCMP	C_9_H_14_N_3_O_7_P	9.71	307.1417	1.44801	0.0138077	1.76661	0.00637104	0.508715934

**Table 2:** Potential biomarkers in feces treated by CAN.

No.	Metabolites	Formula	Rt (min)	*m*/*z*	VIP	CON vs. MOD		MOD vs. SA	
						*p*	FC	*p*	FC
1	Nicotinic acid	C_6_H_5_NO_2_	9.89	123.1175	1.67863	0.000608505	3.59901	0.000111543	0.144503
2	Indoleacetic acid	C_10_H_9_NO_2_	18.83	175.146	1.5584	0.00247057	0.529392	0.0365076	1.33883
3	Tyrosine methylester	C_10_H_13_NO_3_	7.17	195.0636	1.33353	0.0159653	1.90626	0.00435013	0.414093
4	Sphingosine	C_18_H_37_NO_2_	16.23	299.2932	1.37008	0.0143366	1.31612	0.00198898	0.597212
5	Arachidonic acid	C_20_H_32_O_2_	6.79	304.1652	1.80389	4.04 × 10^−5^	3.04138	1.16567 × 10^−5^	0.277048
6	Eicosadienoic acid	C_20_H_36_O_2_	0.73	308.2173	1.3962	0.0105847	1.78669	1.07487 × 10^−6^	0.274185
7	N2,N2-Dimethylguanosine	C_12_H_17_N_5_O_5_	5.62	311.1033	1.21087	0.0351331	0.681728	0.00648757	1.27209
8	Alpha-linolenoyl ethanolamide	C_20_H_35_NO_2_	8.49	321.182	1.43032	0.00842418	0.575453	1.29437 × 10^−9^	1.90028
9	Cholic acid	C_24_H_40_O_5_	12.99	408.3122	1.32594	0.0170507	1.56328	0.0163017	0.651426
10	Lanosterol	C_30_H_50_O	16.52	426.3427	1.62204	0.00124937	0.627569	0.00501348	1.26061
11	Cytidine triphosphate	C_9_H_16_N_3_O_14_P_3_	0.9	483.1095	1.78379	6.73 × 10^−5^	3.97233	8.72003 × 10^−5^	0.269265
12	Creatinine	C_4_H_7_N_3_O	6.53	112.9857	1.70469	0.00146147	0.422791	0.00420806	1.40028
13	Glutathione	C_10_H_17_N_3_O_6_S	2.99	306.9867	1.68886	0.00174648	3.66615	0.00447052	0.319507
14	dADP	C_10_H_15_N_5_O_9_P_2_	9.94	411.102	1.51041	0.00874459	0.623059	0.000188725	2.44321
15	Glycocholic acid	C_26_H_43_NO_6_	10.82	465.2788	1.31837	0.0299595	0.613165	0.000233495	1.9087

Moreover, 15 metabolites were identified in the CON, MOD, and SA groups (Figures 6E–G). Compared to the CON group, the MOD group had significantly upregulated levels of nicotinic acid, tyrosine methylester, sphingosine, arachidonic acid, eicosadienoic acid, cholic acid, cytidine triphosphate, and glutathione, whereas indoleacetic acid, N2,N2-dimethylguanosine, alpha-Linolenoyl ethanolamide, lanosterol, creatinine, dADP, and glycocholic acid were downregulated. These metabolites returned to normal levels after AN and CAN treatments, indicating the effective improvement of metabolic disorders in constipated rats by AN and CAN.

### 3.7 Metabolic pathway analysis

**Figure 7:**
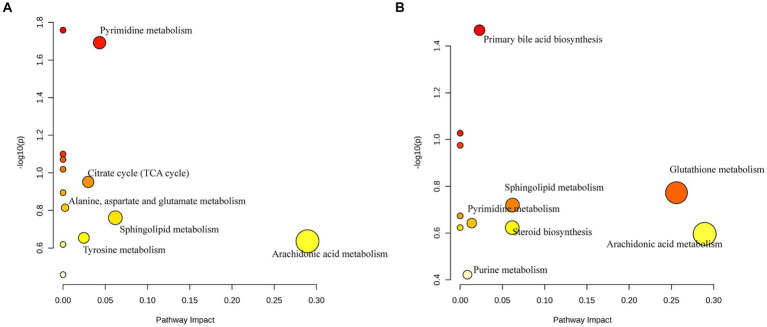
Enrichment analysis of potential metabolic pathways in **(A)** AN and **(B)** SA rat’s feces.

The identified metabolites were imported into the MetaboAnalyst database for potential metabolic pathway analysis. The data revealed that AN-treated metabolites were associated with arachidonic acid, sphingolipid, pyrimidine, tyrosine, alanine, aspartate, and glutamate metabolisms, as well as TCA cycle (Figure 7A). Whereas the CAN-treated metabolites were associated with arachidonic acid, glutathione, sphingolipid, pyrimidine, and purine metabolisms, as well as steroid and primary bile acid biosynthesis (Figure 7B). Among these, arachidonic acid, sphingolipid, and pyrimidine metabolisms were metabolic pathways regulated by both AN and CAN.

### 3.8 The correlation analysis between gut microbiota and metabolites

**Figure 8:**
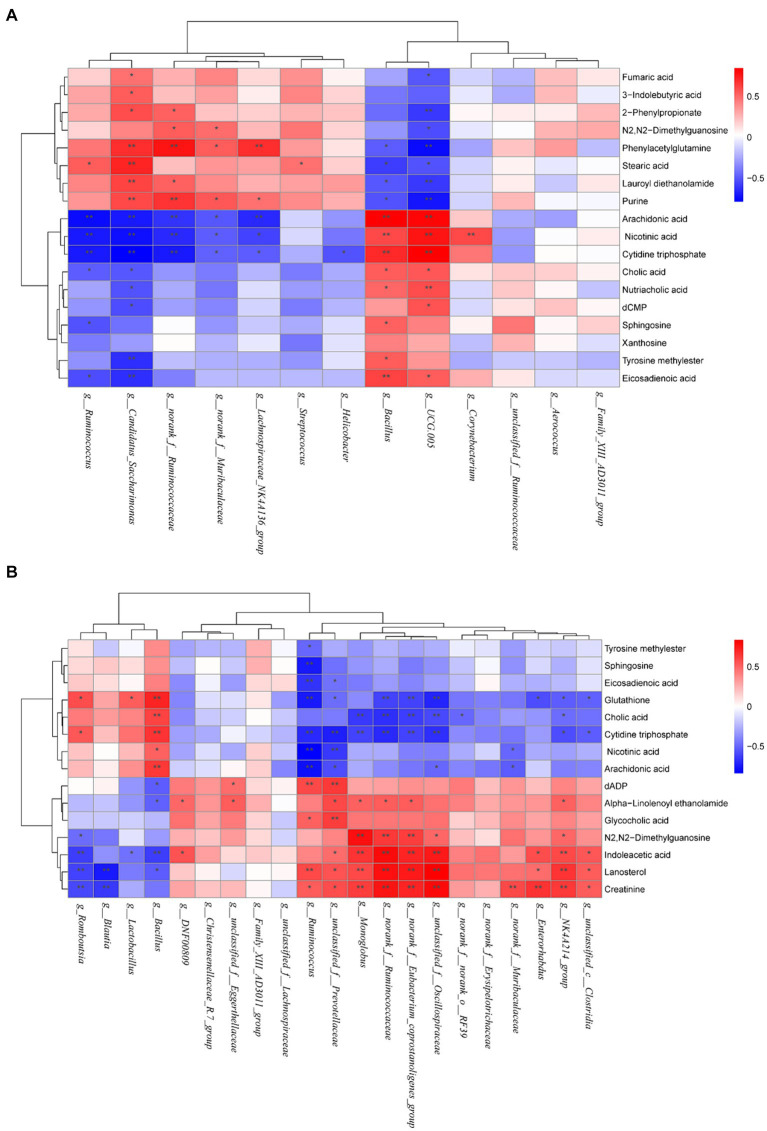
Heatmaps of correlation between gut microbiota and differential metabolites in **(A)** CON, MOD, and AN groups as well as **(B)** in CON, MOD, and SA groups. Red represents positive correlation; blue represents negative correlation, and the darker the color, the greater the correlation coefficient. ^*^p < 0.05 and ^**^p < 0.01.

The correlation between different metabolites and intestinal flora in CON, MOD, and AN groups was analyzed, which indicated significant correlations between differential metabolites and differential bacterial genera including *g_Bacillus*, *g_UCG-005*, *g_Candidatus_Saccharimonas*, *g_norank_f_Ruminococcaceae* (Figure 8A). *g_Bacillus* and *g_UCG-005* genera showed negative correlations with metabolites phenylacetylglutamine, stearic acid, lauroyl diethanolamide, and purine, whereas indicated positive correlations with arachidonic acid, nicotinic acid, cytidine triphosphate, nutriacholic acid, cholic acid, and eicosadienoic acid. Furthermore, *g_Candidatus_Saccharimonas* and *g_norank_f_Ruminococcaceae* genera were positively correlated with the following metabolites: phenylacetylglutamine, purine, 2-phenylpropionate, and lauroyl diethanolamide, whereas negatively correlated with arachidonic acid, nicotinic acid, and cytidine triphosphate.

**Figure 9:**
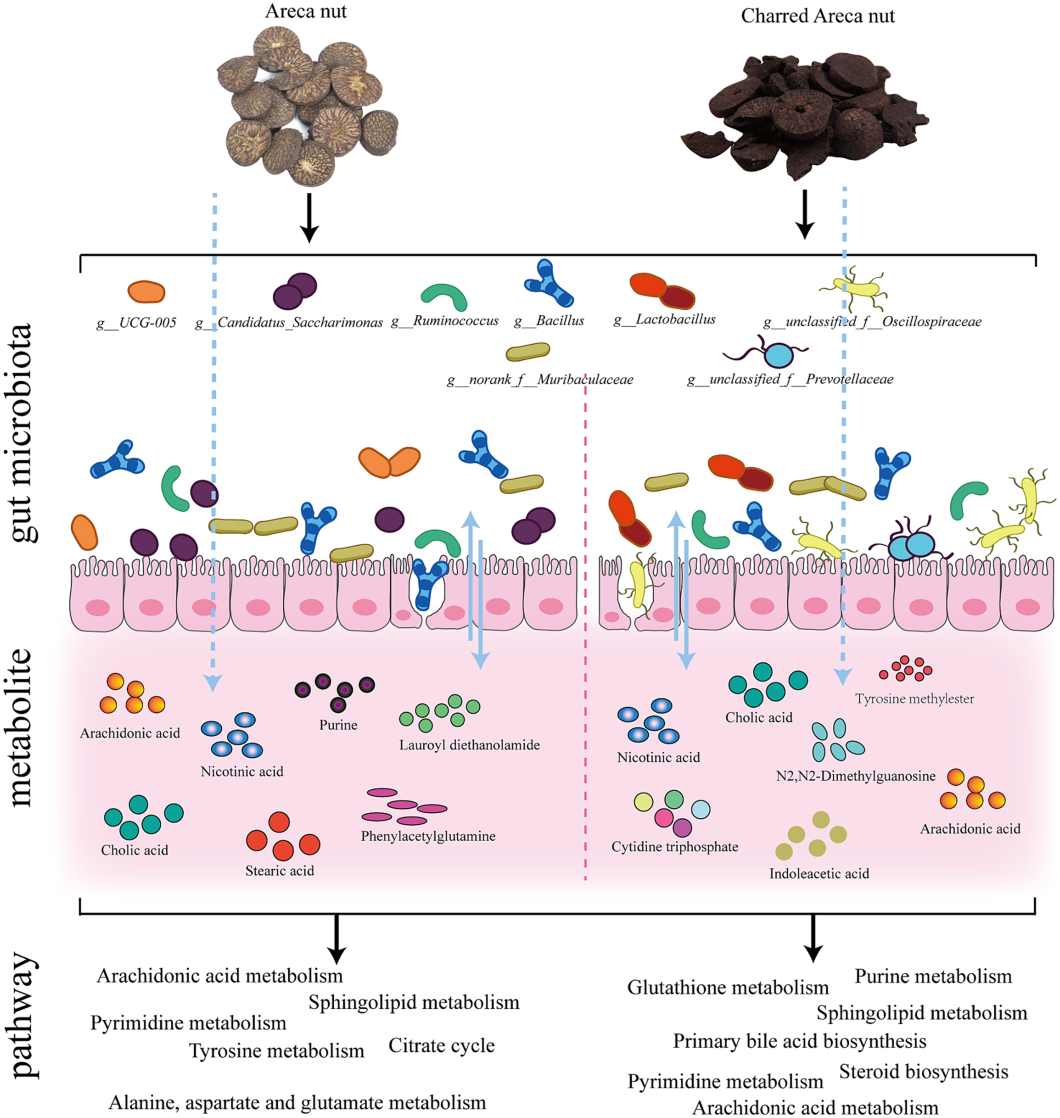
Mechanisms of AN and CAN.

The correlation analysis of differential metabolites and intestinal microbiota between the CON, MOD, and SA groups revealed that significantly correlated differential bacterial genera including *g_Bacillus*, *g_Romboutsia*, *g_norank_f_Eubacterium_coprostanoligenes_group*, *g_unclassified_f_Oscillospiraceae*, *g_Monoglobus*, *g_NK4A214_group*, *g_Enterorhabdus*, *g_norank_f_Ruminococcaceae*, *g_Ruminococcus*, and *g_unclassified_f_Prevotellaceae*. *g_Romboutsia* and *g_Bacillus* were positively linked with glutathione, and cytidine triphosphate and negatively correlated with indoleacetic acid, lanosterol. (Figure 8B). *g_unclassified_f_Oscillospiraceae*, *g_norank_f_Eubacterium_coprostanoligenes_group*, *g_norank_f_Ruminococcaceae* and *g_NK4A214_group* exhibited positive correlations with N2,N2-dimethylguanosine, indoleacetic acid, lanosterol and creatinine, and negative correlations with glutathione, cholic acid and cytidine triphosphate. Furthermore, *g_Enterorhabdus* showed positive correlations with indoleacetic acid, lanosterol, and creatinine and negative correlations with glutathione. Moreover, *g_Monoglobus* showed positive correlations with N2,N2-dimethylguanosine, indoleacetic acid, lanosterol, and creatinine and negative correlations with cholic acid and cytidine triphosphate. While *g_Ruminococcus* and *g_unclassified_f_Prevotellaceae* were positively correlated with eicosadienoic acid, glutathione, cytidine triphosphate, nicotinic acid, and arachidonic acid, and negatively correlated with dADP, glycocholic acid, lanosterol, and creatinine (see Figure 9).

## 4 Discussion

Loperamide is one commonly used for establishing animal constipation models for research studies. It has been observed to inhibit colon longitudinal activity and circular muscle pressure, reduce propulsive activity, and increase non-propulsive activity, thereby prolonging intestinal transit time. The use of loperamide under normal circumstances can lead to GI motility disorders, resulting in constipation (32, 33).

This study employed loperamide to induce constipation symptoms in rats, including slow weight gain as well as substantially reduced intestinal propulsion rate and fecal water content. However, AN and CAN interventions effectively ameliorated these symptoms, indicating their potential to enhance GI motility and facilitate defecation. The literature suggests that the intestinal barrier is crucial for maintaining gut health, and its disruption can affect intestinal motility and frequency. In the colon, the mucosal barrier contains goblet cells-secreted antimicrobial peptides, which defend against microbial invasion and maintain tissue homeostasis (34, 35). In this research, hematoxylin and eosin staining revealed that AN and CAN effectively alleviate constipation-induced damage to the colonic mucosa, crypt structures, and muscular layers. These findings confirm that both AN and CAN could effectively improve rats’ impaired intestinal motility and intestinal barrier damage. Furthermore, GI motility-related hormones, including MTL, VIP, SP, and ACh, are crucial in regulating GI movement. MTL can regulate smooth muscle contraction, promote gastric protease secretion, and improve GI motility disorders (36). Substance P is an excitatory neuropeptide that has been observed to promote GI peristalsis, thereby preventing constipation caused by rectal sensory dysfunction (37). Moreover, VIP is a peptide hormone and intestinal vasodilator that stimulates colonic motility (38). ACh promotes defecation by inducing intestinal contractions to alleviate constipation (39). Overall, this investigation revealed that AN and CAN interventions could improve the reduced serum levels of MTL, SP, VIP, and ACh, thus alleviating constipation.

Several studies have indicated that gut microbiota is correlated with constipation (17). Here, alpha diversity analysis revealed that CAN alleviated the loperamide-induced decrease in species abundance, thereby increasing community diversity. Furthermore, the NMDS plot of beta diversity analysis indicated alterations in the structure of intestinal microbial communities in constipated rats, suggesting that AN and CAN altered the intestinal microbial community structure to varying degrees in constipated rats. Previous studies have revealed that Bacteroidetes can promote energy production and transformation by digesting dietary fiber polysaccharides (40). Moreover, compared to healthy rats, constipated model rats have notably increased intestinal F/B ratio (41). Alterations in this ratio may accelerate energy intake, leading to obesity (42), as observed in obese children with relatively decreased gut Bacteroidetes abundance (43). These data are consistent with the current study, where the Firmicutes/Bacteroides ratio in constipated rat’s intestines was significantly increased compared to healthy rats; however, AN and CAN treatments effectively reversed this effect. In addition, it was observed that the abundance change of Bacteroidetes was more significant, which significantly decreased in the MOD group but was alleviated by AN and CAN treatments. At the phylum level, the relative abundance of Proteobacteria decreased after loperamide-induced constipation, which was effectively improved by intervention with AN and CAN. At the genus level, the constipated rat’s gut microbiota had decreased abundance of *g_norank_f_Muribaculaceae*, *g_Ruminococcus*, *g_Candidatus_Saccharimonas*, *g_unclassified_f_Oscillospiraceae*, and *g_unclassified_f_Prevotellaceae*, while the AN and SA rats indicated increased *g_Bacillus*, *g_UCG-005*, and *g_Lactobacillus* abundance to varying degrees of recovery. In summary, the structure of the gut microbiota was disrupted after loperamide induction in rats; however, this disruption was alleviated by AN and CAN.

Intestinal microbiota and host-produced metabolites are beneficial for maintaining intestinal microenvironment stability and normal physiological functions (44). Metabolomic analysis of rat’s feces indicated significant changes in 25 metabolites following loperamide induction, including nicotinic acid, tyrosine methylester, sphingosine, arachidonic acid, eicosadienoic acid, N2,N2-dimethylguanosine, cholic acid, cytidine triphosphate, 2-phenylpropionate, phenylacetylglutamine, stearic acid, lauroyl diethanolamide, nutriacholic acid, fumaric acid, purine, 3-indolebutyric acid, xanthosine, dCMP, indoleacetic acid, alpha-Linolenoyl ethanolamide, lanosterol, creatinine, glutathione, dADP, and glycocholic acid. Furthermore, it was observed that AN and CAN restored fecal metabolites, suggesting that they can alter the metabolic profile by regulating fecal metabolites, thereby improving intestinal function and alleviating constipation symptoms. Moreover, the 10 most enriched metabolic pathways included arachidonic acid, sphingolipid, and pyrimidine metabolisms (associated with both AN and CAN), whereas citrate cycle (TCA cycle) along with tyrosine, alanine, aspartate, and glutamate metabolisms were enriched by AN, whereas glutathione and purine metabolism metabolisms, as well as steroid and primary bile acid biosynthesis pathways were enriched by CAN.

Arachidonic acid is primarily metabolized by three enzymes: cytochrome P450, lipoxygenase, and cyclooxygenase, thereby producing leukotrienes, thromboxanes, and prostaglandins, the three inflammatory mediators that induce inflammation (45). Inflammation has been observed to disrupt the intestinal barrier integrity, which results in decreased excitatory neurotransmitters and hormone secretion, thereby inhibiting GI motility and stimulating constipation (46, 47). In this study, arachidonic acid levels were significantly elevated in loperamide-induced constipated rats; however, these levels were markedly reduced after AN and CAN interventions, leading to increased secretion of excitatory neurotransmitters and alleviation of intestinal barrier damage. Research has indicated that sphingolipid metabolism is associated with regulating immune function and inflammatory responses (48). Whereas pyrimidine metabolism is associated with pyrimidine nucleotide synthesis, where cytidine triphosphate and dCMP are intermediate products of pyrimidine synthesis. Moreover, aberrant pyrimidine metabolism has been associated with various diseases, such as immune-related diseases, neurological disorders, and circulatory system diseases (49, 50). Therefore, it is suggested that maintaining pyrimidine metabolism homeostasis can effectively treat certain immune-mediated inflammatory diseases (51, 52). Here, the levels of cytidine triphosphate and dCMP from the fecal pyrimidine metabolism were abnormal in the constipated rats, suggesting the disruption of fecal pyrimidine metabolism, which may promote intestinal inflammation and functional disorders. However, intervention with AN and CAN restored the fecal levels of cytidine triphosphate and dCMP metabolites. Altogether, these data suggested that both AN and CAN can alleviate intestinal inflammation as well as improve intestinal barrier damage and functional disorders in constipated rats by modulating the metabolisms of arachidonic acid, sphingolipid, and pyrimidine.

The TCA cycle, as well as tyrosine, alanine, aspartate, and glutamate metabolisms, play important physiological and nutritional roles. The TCA cycle serves as a crucial hub for lipid, carbohydrate, and amino acid metabolism and provides efficient energy by the oxidation of substances such as glucose (53). Intestinal amino acid metabolism has been observed to be associated with protein synthesis and provide nutrients to intestinal cells as well as maintain their normal physiological functions, such as promoting intestinal mucus secretion, repairing damaged intestinal mucosa, and regulating bacterial barriers and immune functions (54, 55). This suggests that AN provides energy to the intestines and increases intestinal motility by modulating the metabolic imbalance of the TCA cycle as well as tyrosine, alanine, aspartate, and glutamate metabolisms.

Glutathione is a cellular antioxidant that maintains the homeostasis of glutathione metabolism, thereby alleviating intestinal oxidative stress (56). Intestinal motility requires a large amount of energy; therefore, the modulation of energy metabolism in the intestine is crucial for alleviating constipation. Purine metabolism is closely related to energy metabolism, and its metabolites are associated with DNA and RNA synthesis as well as processes such as energy conversion and intracellular signal transduction (57). Primary bile acid biosynthesis has been reported to be essentially linked with GI motility because bile acids promote digestion and absorption in the GI tract (58). Steroid synthesis has not been significantly associated with the occurrence and development of constipation. Here, CAN was observed to reverse the loperamide-induced decrease in steroid biosynthesis markers, such as lanosterol, which is a potential marker for detecting intestinal dysfunction (59) and is closely related to changes in intestinal microbiota and regulation of intestinal inflammatory cytokines (60). These data suggest that CAN may improve constipation by modulating glutathione and purine metabolisms, as well as primary bile acid and steroid biosynthesis, thereby promoting GI energy metabolism, absorption, digestion, and regulation of inflammatory factors.

This study performed Spearman correlation analysis, which revealed that under CAN treatment, there were significant associations between intestinal bacterial genera and fecal metabolites, including *g_norank_f_Ruminococcaceae*, *g_Candidatus_Saccharimonas*, *g_UCG-005*, *g_Bacillus* under AN treatment, and *g_norank_f_Ruminococcaceae*, *g_Enterorhabdus*, *g_Ruminococcus*, *g_NK4A214_group*, g*_norank_f_Eubacterium_coprostanoligenes_group*, *g_Monoglobus*, *g_Romboutsia*, *g_Bacillus*, and *g_unclassified_f_Prevotellaceae*. These bacterial taxa were closely associated with metabolites. The literature suggests that *g_Candidatus_Saccharimonas* are beneficial bacteria that essentially maintain normal intestinal function by enhancing intestinal immunity and maintaining the intestinal barrier (61). Furthermore, *g_norank_f_Ruminococcaceae* and *g_Ruminococcus* have been reported to promote short-chain fatty acids synthesis, which have anti-obesity, neuroprotective, and anti-inflammatory effects (62, 63). Moreover, *g_UCG-005* can also produce a short-chain fatty acid called butyrate; however, it inhibits the contractions of intestinal smooth muscle, interferes with fluid transport in the colon, and induces constipation (64). *g_Enterorhabdus* can hydrolyze protobile acids, promote bile acids excretion, and increase the levels of secondary bile and short-chain fatty acids, thereby delaying inflammation and maintaining intestinal homeostasis (65). *g_norank_f_Eubacterium_coprostanoligenes_group* may reduce cholesterol, lipid, and bile acid metabolism, thereby maintaining colonic barrier function and reducing systemic inflammation (66). Studies have indicated that *g_Monoglobus* can effectively degrade pectin in the human colon; however, its abundance is positively correlated with blood ammonia levels, and high ammonia levels can disrupt the intestinal epithelial barrier, which is an inflammatory pathogenesis of chronic kidney disease (67). *g_Bacillus* comprises various pathogenic bacteria and produces enterotoxins (68). Previous research has revealed that the abundance of *g_Romboutsia* is abnormally elevated in various diseases such as irritable bowel syndrome, ulcerative colitis, and depression (69). Based on these findings, it can be inferred that AN and CAN may regulate constipation via intestinal bacteria and their metabolites, as well as their correlation.

In summary, this study revealed that both AN may protect GI mucosa and enhance GI motility, and alleviate constipation symptoms by regulating the relative abundance of specific gut microbiota (*Bacillus*, *UCG-005*, *norank_f__Muribaculaceae*, *Candidatus_Saccharimonas*, *Ruminococcus*) as well as citrate cycle or tyrosine, alanine, aspartate, and glutamate metabolic pathways. Furthermore, CAN was observed to promote gastric emptying and intestinal propulsion, thereby alleviating constipation, by modulating the relative abundance of specific gut microbiota (*Lactobacillus*, *Bacillus*, *norank_f__Muribaculaceae*, *Ruminococcus*, *unclassified_f__Oscillospiraceae*, *unclassified_f__Prevotellaceae*) along with steroid and primary bile acid biosynthesis, as well as pyrimidine and purine metabolic pathways. These data indicated that both AN and CAN improved constipation however, their underlying mechanisms of action were not the same. After processing, CAN promoted a stronger therapeutic effect against constipation via a new mechanism, suggesting that compared with non-processed health food, the processing of health food can significantly enhance its mechanism of action. This study provides novel theoretical evidence for the subsequent study of the changes in pharmacological activity caused by the processing of natural medicines.

## Data Availability

The original contributions presented in this study are included in the article/Supplementary material; further inquiries can be directed to the corresponding author.

## Footnotes

^1^ https://cloud.majorbio.com

^2^ http://www.hmdb.ca/

^3^ http://www.kegg.ca/

^4^ http://www.metaboanalyst.ca/

## Supplementary material

SUPPLEMENTARY FIGURE S1 Rarefaction curves included **(A)** Shannon index and **(B)** Coverage index. **(C)** Venn diagram of the five groups. The alpha diversity analysis included the **(D)** Ace index, **(E)** Chao index, **(F)** Simpson index, and **(G)** Sobs index. **(H)** NMDS. ^#^*p* < 0.05 and ^##^*p* < 0.01 vs. CON group.

SUPPLEMENTARY FIGURE S2 AN and CAN altered the microbial composition at the phylum levels. **(A)** Microbial community composition at the phylum level. **(B)** The ratio of Firmicutes to Bacteroidetes at the phylum level. **(C)** Relative abundance of Bacteroidetes and **(D)** Proteobacteria phyla. Data are displayed as mean ± SD. ^###^*p* < 0.001 vs. CON group. ^*^*p* < 0.05, ^**^*p* < 0.01, and ^***^*p* < 0.001 vs. MOD group.

SUPPLEMENTARY FIGURE S3 LEfSe cladogram (circles from outer to inner represent genus, family, order, class, and phylum).

SUPPLEMENTARY FIGURE S4 PCA, OPLS-DA analysis of rat feces sample. **(A)** PCA diagram in positive ion mode. **(B)** PCA diagram in negative ion mode. **(C,E,G)** OPLS-DA diagram in positive ion mode. **(D,F,H)** OPLS-DA diagram in negative ion mode.

SUPPLEMENTARY FIGURE S5 Permutation test analysis of rat’s feces sample. **(A,C,E)** Permutation test diagram in positive ion mode. **(B,D,F)** Permutation test diagram in negative ion mode.

SUPPLEMENTARY FIGURE S6 Volcano gram analysis of differential metabolites in feces sample. **(A)** CON vs. MOD in positive and **(B)** negative ion modes. **(C)** MOD vs. AN in positive and **(D)** negative ion modes. **(E)** MOD vs. SA in positive and **(F)** negative ion modes.
