# Correction: Metabolomics and gut microbiota analysis reveal the differential efficacy of areca nut and charred areca nut in treating constipation

**DOI:** 10.3389/fnut.2025.1713576

**Published:** 2025-11-11

**Authors:** Li-sha Wang, Jiao-xia Wu, Fang Zhang, Yan Huang, Yue-xia Jiang, Yong-hui Li

**Affiliations:** Key Laboratory of Tropical Translational Medicine of Ministry of Education, Hainan Provincial Key Laboratory of Research and Development on Tropical Herbs, Haikou Key Laboratory of Li Nationality Medicine, The Second Affiliated Hospital of Hainan Medical University, School of Pharmacy, Hainan Medical University, Haikou, China

**Keywords:** areca nut, charred areca nut, constipation, gut microbiota, metabolomics

There was a mistake in the **Funding** statement as published. An incorrect grant number was provided for Hainan Provincial Natural Science Foundation of China (“SZR220007”). The correct grant number is “822MS073”.

The correct **Funding** statement reads:

“The author(s) declare that financial support was received for the research and/or publication of this article. This research was supported by the Hainan Provincial Natural Science Foundation of China (No. 822MS073).”

The original version of this article has been updated.

