# Supplementary figures and images for: Metabolomics and gut microbiota analysis reveal the differential efficacy of areca nut and charred areca nut in treating constipation
Source: Front Nutr. 2024 Sep 13;11:1455824. doi: 10.3389/fnut.2024.1455824 (PMC11427381; doi:10.3389/fnut.2024.1455824)

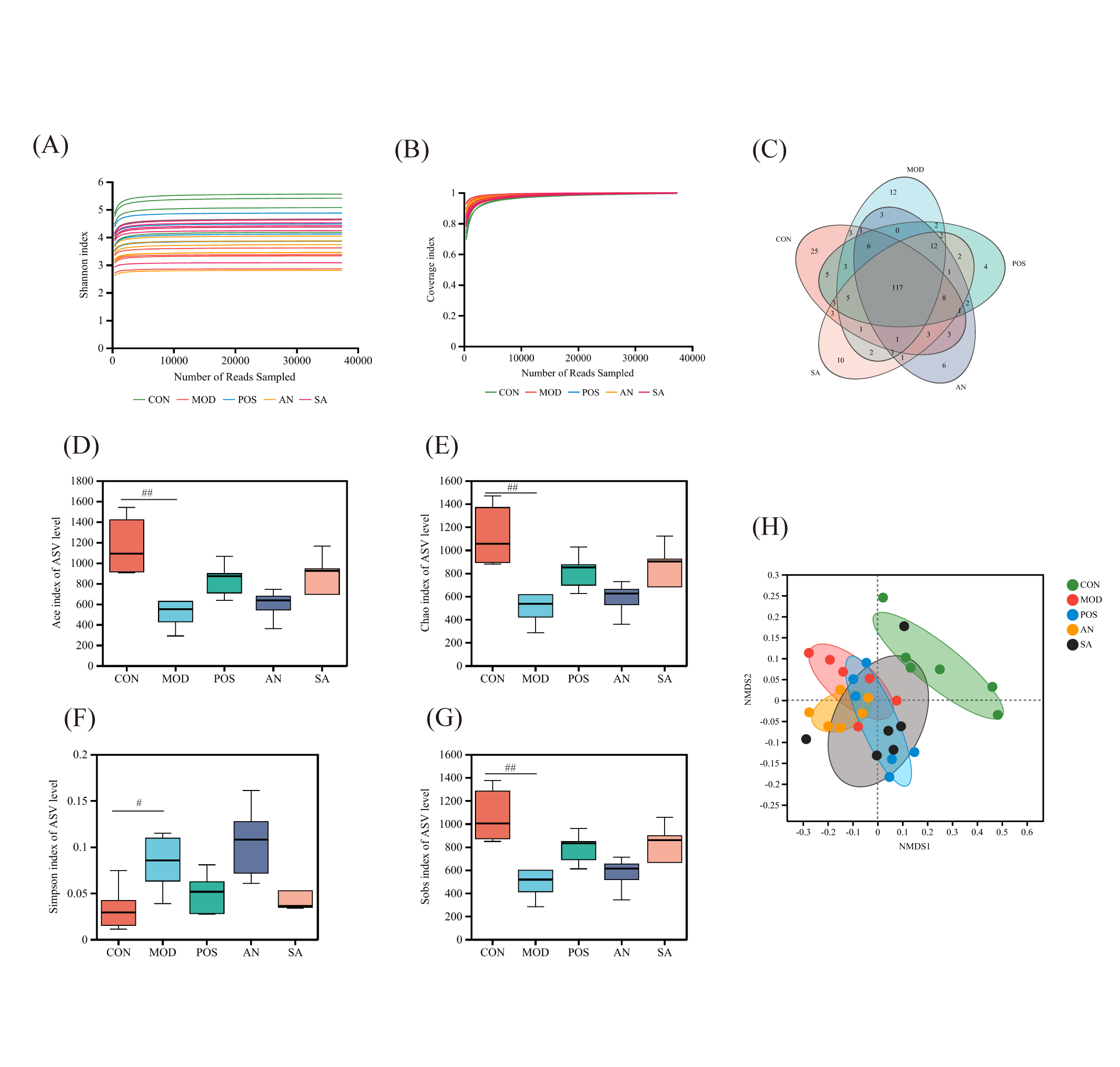

Supplement: SUPPLEMENTARY FIGURE S1 — Rarefaction curves included (A) Shannon index and (B) Coverage index. (C) Venn diagram of the five groups. The alpha diversity analysis included the (D) Ace index, (E) Chao index, (F) Simpson index, and (G) Sobs index. (H) NMDS. #p < 0.05 and ##p < 0.01 vs. CON group. [file Image_1.TIF]

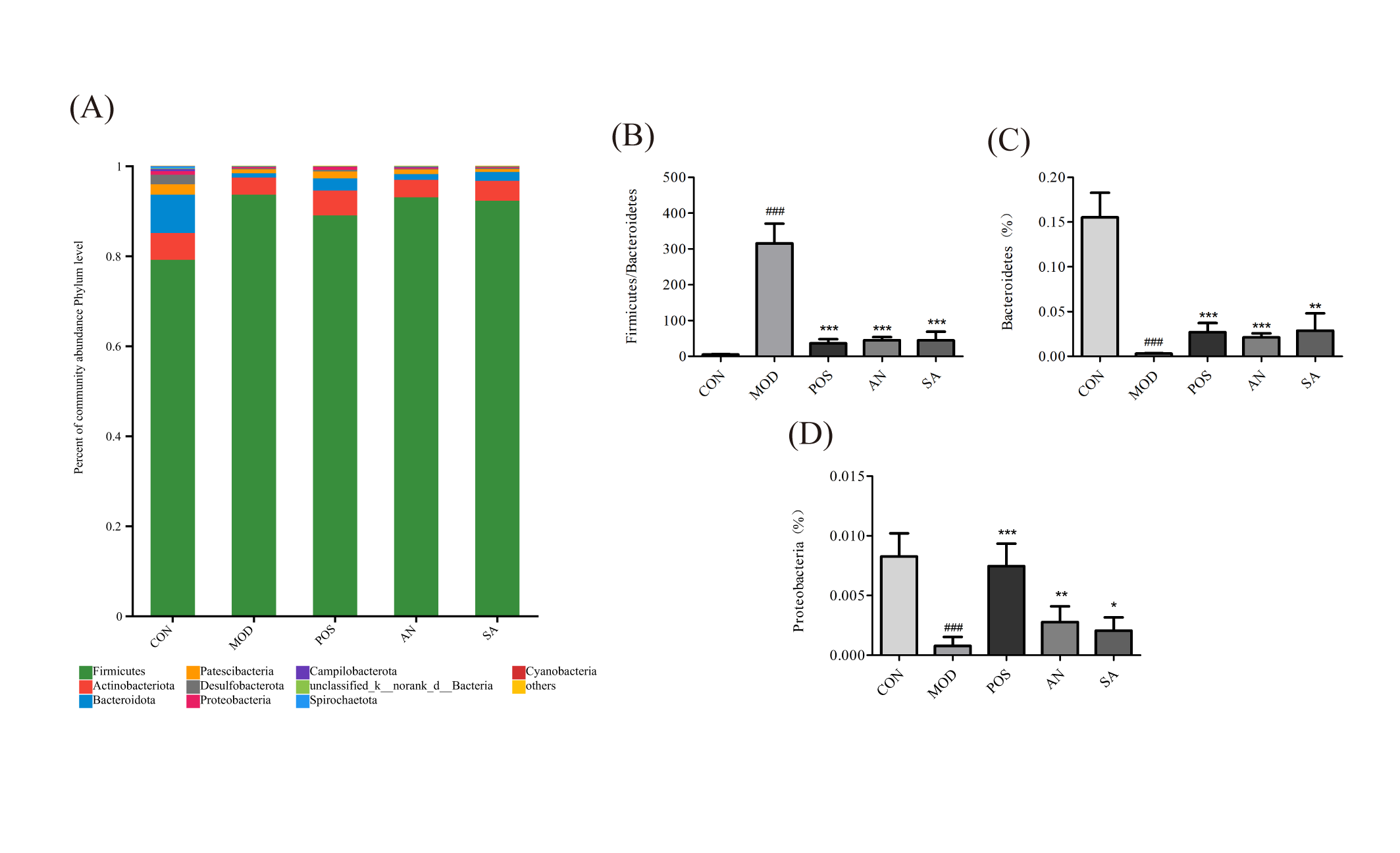

Supplement: SUPPLEMENTARY FIGURE S2 — AN and CAN altered the microbial composition at the phylum levels. (A) Microbial community composition at the phylum level. (B) The ratio of Firmicutes to Bacteroidetes at the phylum level. (C) Relative abundance of Bacteroidetes and (D) Proteobacteria phyla. Data are displayed as mean ± SD. ###p < 0.001 vs. CON group. *p < 0.05, **p < 0.01, and ***p < 0.001 vs. MOD group. [file Image_2.TIF]

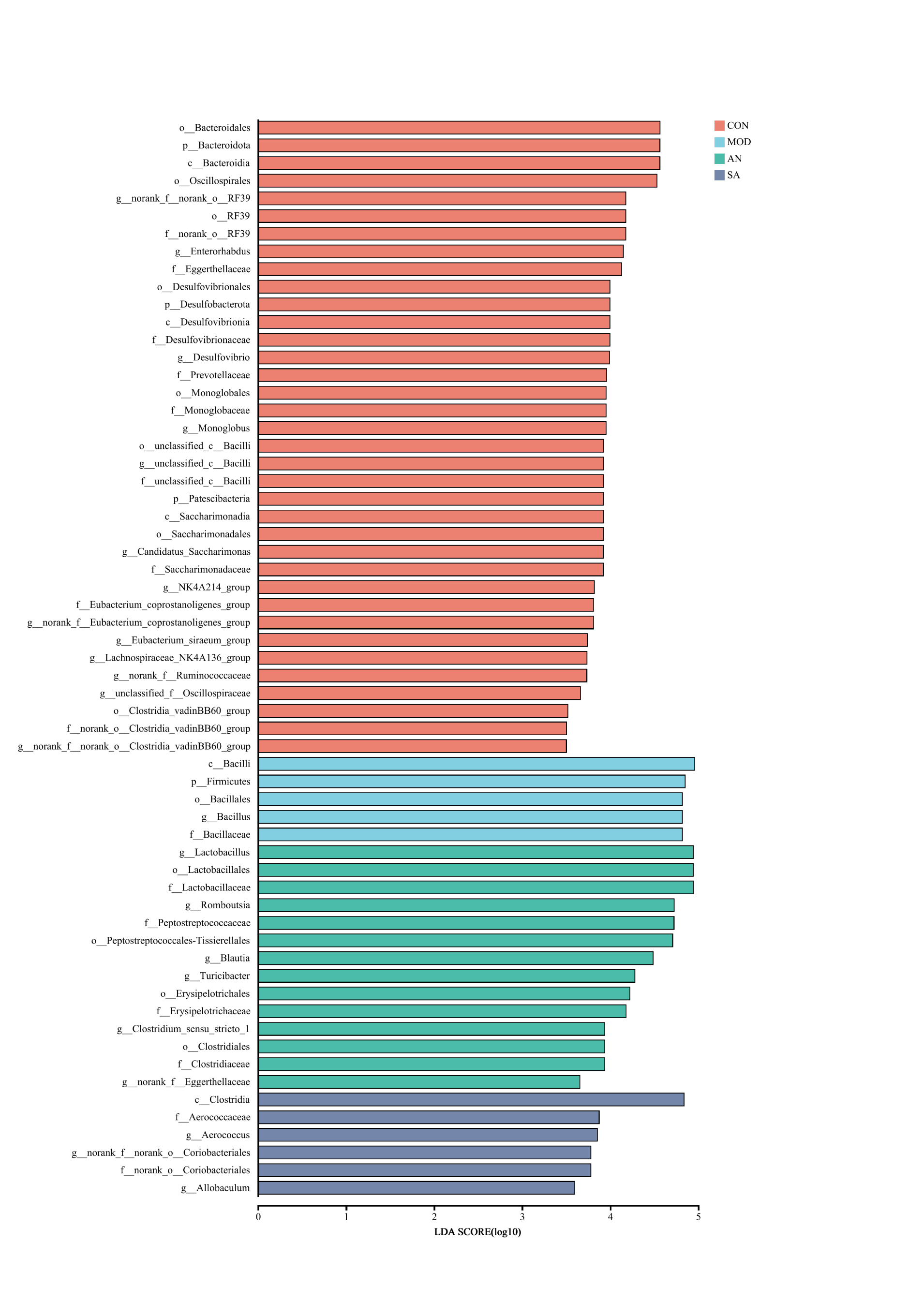

Supplement: SUPPLEMENTARY FIGURE S3 — LEfSe cladogram (circles from outer to inner represent genus, family, order, class, and phylum). [file Image_3.TIF]

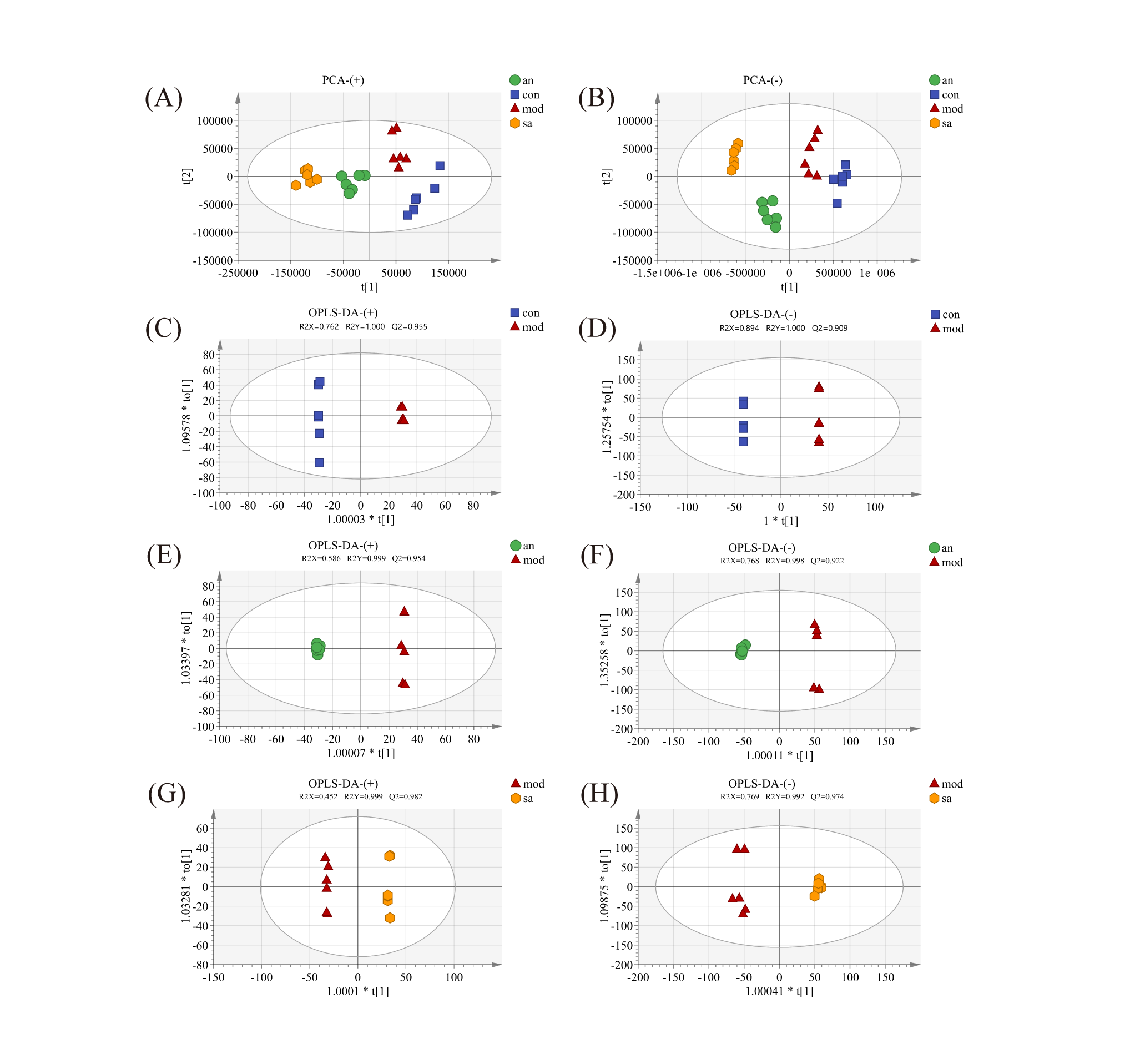

Supplement: SUPPLEMENTARY FIGURE S4 — PCA, OPLS-DA analysis of rat feces sample. (A) PCA diagram in positive ion mode. (B) PCA diagram in negative ion mode. (C,E,G) OPLS-DA diagram in positive ion mode. (D,F,H) OPLS-DA diagram in negative ion mode. [file Image_4.TIF]

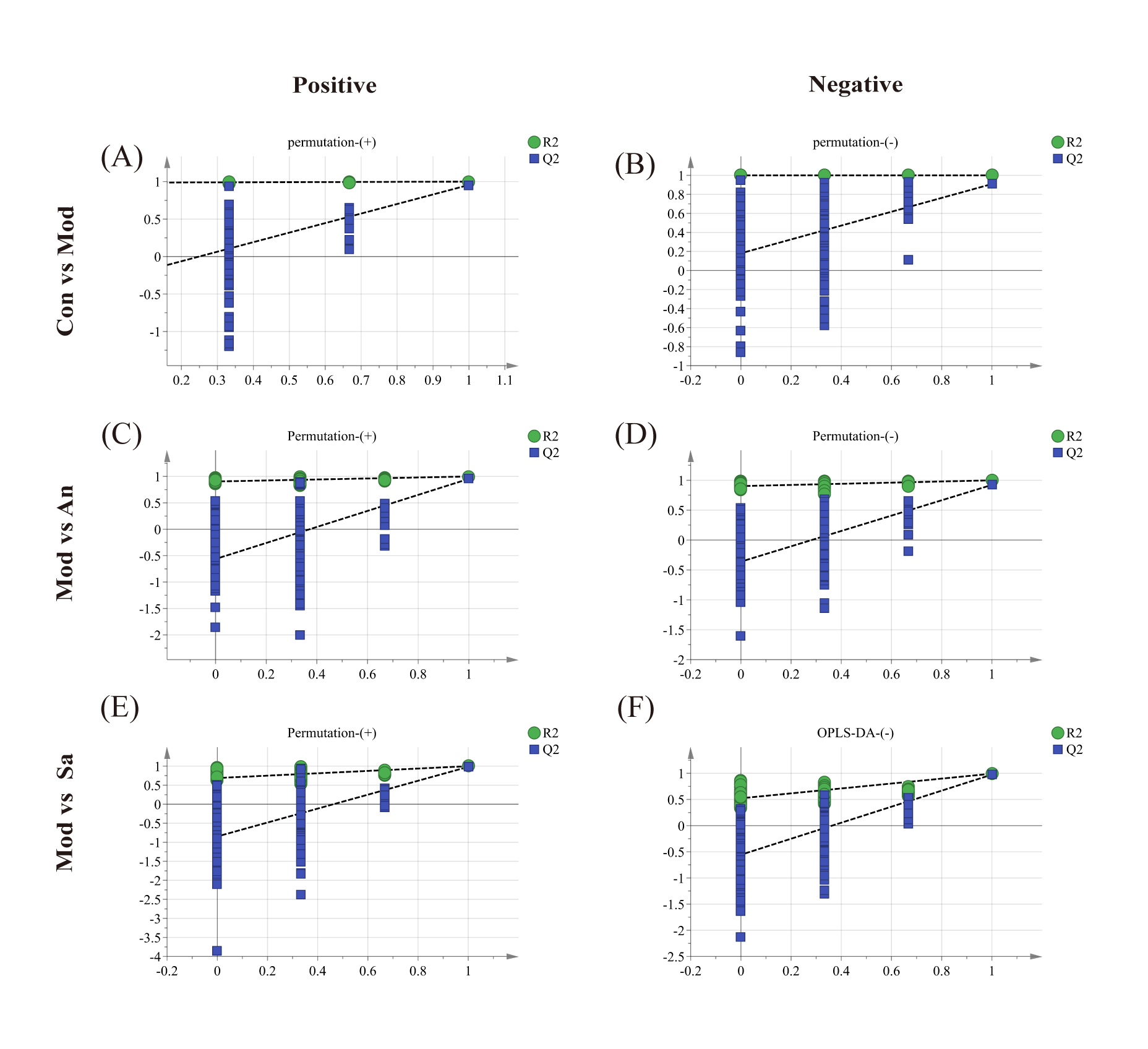

Supplement: SUPPLEMENTARY FIGURE S5 — Permutation test analysis of rat’s feces sample. (A,C,E) Permutation test diagram in positive ion mode. (B,D,F) Permutation test diagram in negative ion mode. [file Image_5.TIF]

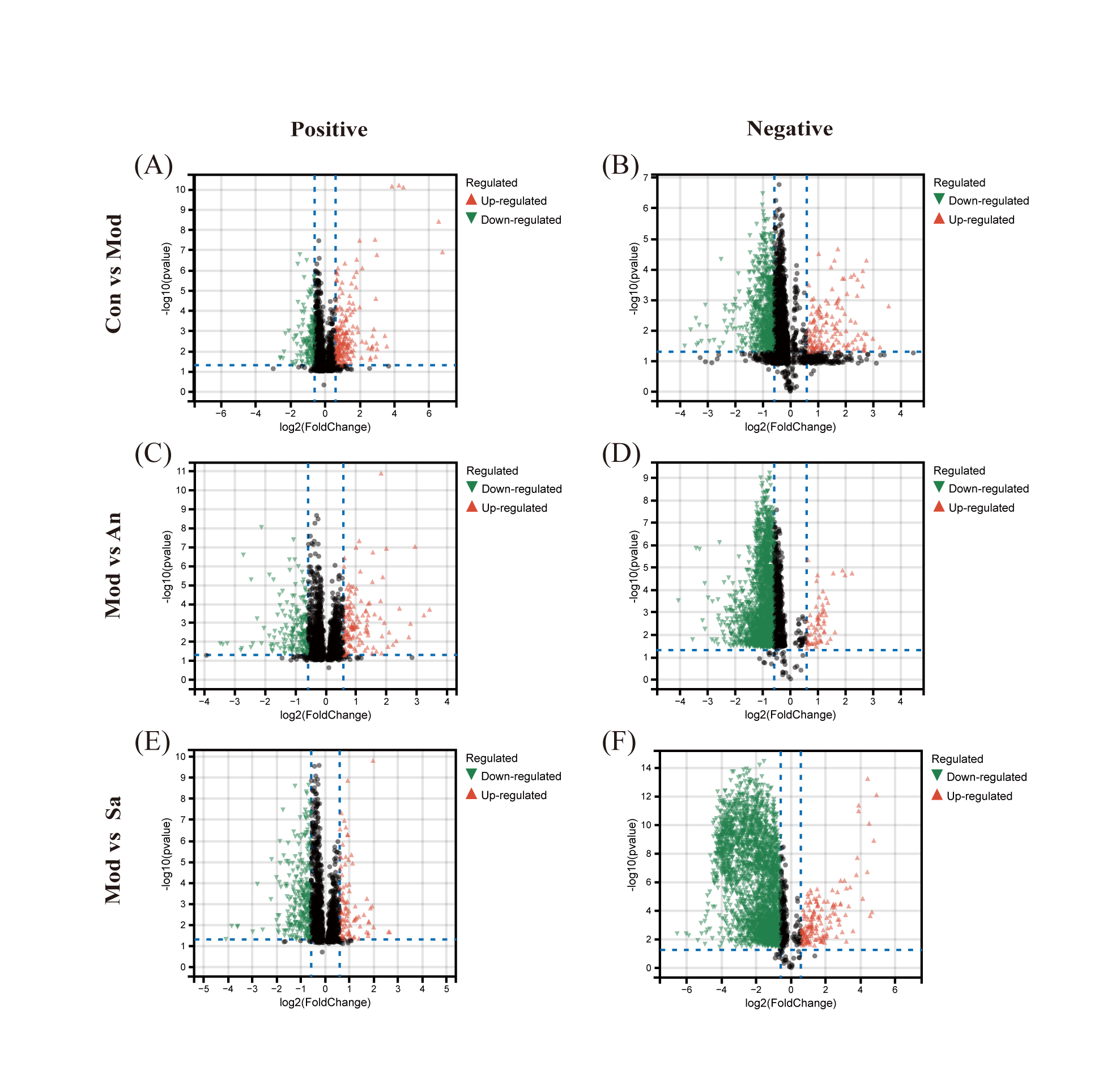

Supplement: SUPPLEMENTARY FIGURE S6 — Volcano gram analysis of differential metabolites in feces sample. (A) CON vs. MOD in positive and (B) negative ion modes. (C) MOD vs. AN in positive and (D) negative ion modes. (E) MOD vs. SA in positive and (F) negative ion modes. [file Image_6.TIF]
